# The Photodynamic Antibacterial Potential of New Tetracationic Zinc(II) Phthalocyanines Bearing 4-((Diethylmethylammonium)methyl)phenoxy Substituents

**DOI:** 10.3390/ijms26199414

**Published:** 2025-09-26

**Authors:** Gennady Meerovich, Dmitry Bunin, Ekaterina Akhlyustina, Igor Romanishkin, Vladimir Levkin, Sergey Kharnas, Maria Stepanova, Alexander Martynov, Victor Loschenov, Yulia Gorbunova, Marina Strakhovskaya

**Affiliations:** 1 Prokhorov General Physics Institute of the Russian Academy of Sciences, Moscow 119991, Russia; gennadymeerovich@gmail.com (G.M.); igor.romanishkin@nsc.gpi.ru (I.R.); loschenov@mail.ru (V.L.); 2 Institute of Engineering Physics for Biomedicine, National Research Nuclear University MEPhI, Moscow 115409, Russia; katya_ahlyustina@mail.ru; 3 Department of Oncology, Radiotherapy and Reconstructive Surgery, Levshin Institute of Cluster Oncology, Sechenov First Moscow State Medical University, Moscow 119435, Russia; levkin_v_v@staff.sechenov.ru (V.L.); kharnas_s_s@staff.sechenov.ru (S.K.); 4 Frumkin Institute of Physical Chemistry and Electrochemistry, Russian Academy of Sciences, Moscow 119071, Russia; bunindm95@gmail.com (D.B.); mpstepanova@edu.hse.ru (M.S.); martynov.alexandre@gmail.com (A.M.); yulia.gorbunova@gmail.com (Y.G.); 5 Faculty of Chemistry, National Research University Higher School of Economics, Moscow 109028, Russia; 6 Kurnakov Institute of General and Inorganic Chemistry, Russian Academy of Sciences, Moscow 119071, Russia; 7 Faculty of Biology, Lomonosov Moscow State University, Moscow 119234, Russia

**Keywords:** photodynamic inactivation, Gram-negative bacteria, tetracationic photosensitizer, aryloxy-substituted Zn(II) phthalocyanines, fluorescence microscopy, bioluminescence, zeta potential

## Abstract

Photodynamic inactivation and antimicrobial photodynamic therapy (PDI/APDT) based on the toxic properties of reactive oxygen species (ROS), which are generated by a number of photoexcited dyes, are promising for preventing and treating infections, especially those associated with drug-resistant pathogens. The negatively charged bacterial cell surface attracts polycationic photosensitizers, which contribute to the vulnerability of the bacterial plasma membrane to ROS. The integrity of the plasma membrane is critical for the viability of the bacterial cell. Polycationic phthalocyanines are regarded as promising photosensitizers due to their high quantum yields of ROS generation (mainly singlet oxygen), high extinction coefficients in the far-red spectral range, and low dark toxicity. For application in PDI/APDT, the wide range of possibilities of modifying the chemical structure of phthalocyanines is particularly valuable, especially by introducing various peripheral and non-peripheral substituents into the benzene rings. Depending on the type and location of such substituents, it is possible to obtain photosensitizers with different photophysical properties, photochemical activity, solubility in an aqueous medium, biocompatibility, and tropism for certain structures of photoinactivation targets. In this study, we tested novel water-soluble Zn (II) phthalocyanines bearing four 4-((diethylmethylammonium)methyl)phenoxy substituents with symmetric and asymmetric charge distributions for photodynamic antibacterial activity and compared them with those of water-soluble octacationic zinc octakis(cholinyl)phthalocyanine. The obtained results allow us to conclude that the studied tetracationic aryloxy-substituted Zn(II) phthalocyanines effectively bind to the oppositely charged cell wall of the Gram-negative bacteria *E. coli*. This finding is supported by data on bacteria’s zeta potential neutralization in the presence of phthalocyanine derivatives and fluorescence microscopy images of stained bacterial cells. Asymmetric substitution influences the aggregation and fluorescent characteristics but has little effect on the ability of the studied tetracationic phthalocyanines to sensitize the bioluminescent *E. coli* K12 TG1 strain. Both symmetric and asymmetric aryloxy-substituted phthalocyanines are no less effective in PDI than the water-soluble zinc octakis(cholinyl)phthalocyanine, a photosensitizer with proven antibacterial activity, and have significant potential for further studies as antibacterial photosensitizers.

## 1. Introduction

The loss of effectiveness in the treatment of infectious diseases due to the development of antimicrobial resistance (AMR) in pathogens results in increased morbidity and mortality, prolonged hospital stays, and financial losses, representing a major public health challenge [1]. Due to the progressive growth of microbial resistance and the limited capacity to develop new antimicrobials, the number of deaths associated with resistant pathogens is predicted to increase to 10 million per year by 2050 [2]. The external structures (cell wall, capsule, exopolysaccharides) and the matrix of biofilms are the “first line” of defense of bacterial cells. Modification of the structure and changes in the physicochemical properties of the cell surface are the most important protective mechanisms that allow bacterial cells to reduce tropism and, consequently, the antibacterial activity of many compounds [3]. In Gram-negative bacteria, cells possess an additional outer asymmetric membrane, which is separated from the cytoplasmic (or plasma) membrane by a periplasmic space with a thin layer of peptidoglycan [4]. The external layer of this outer membrane is composed of tightly packed lipopolysaccharides (LPS) characterized by a high content of negatively charged groups in their core region exposed on the external cell surface. Divalent cations of Mg^2+^ and Ca^2+^ form cross-links between neighboring LPS molecules to stabilize the LPS layer. Beneath this externally charged surface of the outer membrane lies the LPS lipid A layer with saturated acyl chains [5]. The inner layers of the outer membrane and the plasma membrane are composed of phospholipids. However, unlike eukaryotic cells, bacterial plasma membranes are enriched with negatively charged phospholipids. This combination of negatively charged and uncharged layers forms an effective intrinsic permeability barrier for lipophilic and anionic hydrophilic molecules. The entry of small, up to 600 Da [6], polar hydrophilic molecules through the outer membrane occurs mainly via embedded protein channels called porins. Thus, the entry of aminoglycosides [7] with a charge from +3 to +5 by *Escherichia coli* cells is most likely realized through “nonspecific” porins. Organic polycations can competitively interact with divalent cations Mg^2+^ and Ca^2+^, displacing them and thereby destabilizing the outer membrane, which promotes the absorption of other molecules of the polycationic agent [8]. At the same time, for molecules of the hydrophobic cation methylene blue, the pathways of penetration through the lipid bilayer of the LPS membrane and through the porin channel are energetically equivalent [9]. Outer LPS and inner plasma membranes create a difficult-to-surpass barrier for adverse environmental factors, biocides, antibiotics, and antiseptics. Developing drugs that can penetrate both membranes of Gram-negative pathogens to reach intracellular targets is a challenge in medicinal chemistry [6].

Photodynamic inactivation (PDI) and antimicrobial photodynamic therapy (APDT) have demonstrated significant potential in the prevention and treatment of infections, particularly associated with drug-resistant pathogens [10]. The technology is based on the toxic properties of reactive oxygen species (ROS) for bacteria, with the low probability of developing resistance to photodynamic effects. ROS are formed as a result of the transfer of electrons (type I reactions) or energy (type II reactions) to molecular oxygen from photoexcited photosensitizer (PS) molecules in the triplet state. In photodynamic reactions of type I, the primary form of ROS is the superoxide anion radical, which can then react to form extremely active hydroxyl radicals. In photodynamic reactions of type II, the primary form of ROS is singlet oxygen.

Phthalocyanines (Pcs) are considered promising photosensitizers for PDI/APDT due to their high extinction coefficients in the spectral range of 670–800 nm (ε > 10^5^ M^−1^cm^−1^), low dark toxicity, and extensive potential for tuning photophysical properties [11,12,13]. Consequently, the ability of phthalocyanine to generate ROS (mainly singlet oxygen) can be significantly affected by the cation complexing agent. In this regard, the diamagnetic cation Zn^2+^ is most appropriate for ensuring a high quantum yield of singlet oxygen photogeneration due to the “heavy atom effect”, which leads to enhanced S_1_→T_1_ intersystem crossing [14,15]. In addition, Zn^2+^ has been shown to form stable complexes with Pcs exhibiting low toxicity, and its salts are inexpensive compared to many other metals, providing the “heavy atom effect” [16].

The negatively charged bacterial cell surface is attractive to polycationic drugs [17]. Polycationic photosensitizers based on phthalocyanine derivatives, with high molecular weight, are incapable of penetrating the outer LPS membrane of Gram-negative bacteria via porin channels. However, given that the primary target of ROS is the bacterial plasma membrane [18] and the diffusion radius of singlet oxygen is comparable to the thickness of the cell wall of Gram-negative bacteria, such binding to the bacterial cell surface can be suitable for effective PDI. Notably, Gram-negative bacteria are able to modify LPS [19], resulting in a reduction in the density of negative charges on the cellular surface and weakening of the interaction with cationic drugs. When synthesizing new antibacterials, this cell surface-associated mechanism of drug resistance requires a balance between the number of positively charged groups, their size and polarity, and the distribution of charges.

Evidence suggests that photosensitizers with asymmetric charge distribution exhibit higher photoactivity than their counterparts with symmetric charge distribution [20,21]. In the present work, we describe the photodynamic antibacterial potential of novel water-soluble Zn (II) phthalocyanines bearing four 4-((diethylmethylammonium)methyl)phenoxy substituents with symmetric and asymmetric charge distribution and compare it with that of water-soluble octacationic zinc octakis(cholinyl)phthalocyanine.

## 2. Results and Discussion

### 2.1. Dye Fluorescence

**Figure 1:**
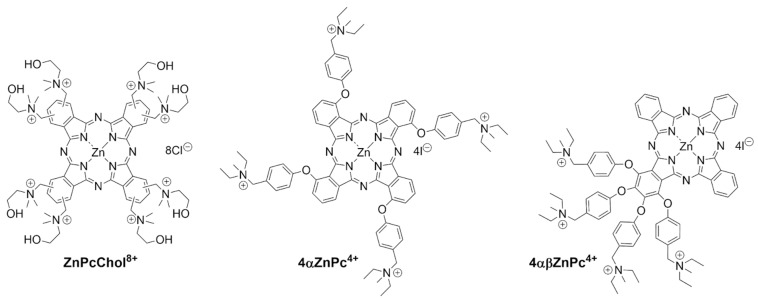
Structures of the phthalocyanine derivatives used in this study.

Previously, we described the preparation and properties of a number of cationic Pcs, including octacationic ZnPcChol^8+^ [22] and tetracationic 4αZnPc^4+^ and 4αβZnPc^4+^ [23,24] with different arrangements of cationic 4-((diethylmethylammonium)methyl)phenoxy-groups (Figure 1).

In 4αZnPc^4+^, aryloxy substituents are symmetrically distributed at nonperipheral positions. In 4αβZnPc^4+^, all four aryloxy substituents are grafted to one of the benzene rings, i.e., two of them are at nonperipheral and two at peripheral positions. Nonperipheral substitution promotes a decrease in aggregation when phthalocyanine derivatives are present in aqueous solutions [25]. In [23], the photophysical and photochemical parameters of cationic Pcs were studied by dissolving these compounds in DMSO and subsequently transferring them into a PBS buffer. In DMSO, 4αZnPc^4+^ and 4αβZnPc^4+^ had high molar absorbance values of the Q-bands, indicating monomeric states of both phthalocyanine derivatives. In accordance with this, in DMSO, 4αZnPc^4+^ and 4αβZnPc^4+^ exhibited high quantum yields of singlet oxygen photogeneration, with Φ_Δ_ values of 0.69 and 0.65, respectively, demonstrating the high photosensitization ability of both compounds. Given the limited water solubility of 4αZnPc^4+^ and 4αβZnPc^4+^, in this study, in the experiments with bacterial cells, we used stock solutions of 4αZnPc^4+^ and 4αβZnPc^4+^ in DMSO. When phthalocyanines were transferred in PBS, DMSO was present in probes in safe biocompatible concentrations [26], which were not more than 0.05%.

**Figure 2:**
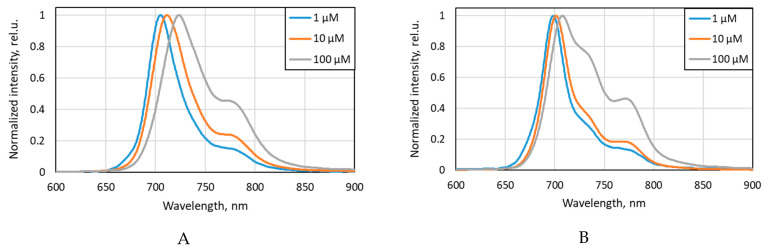
Spectral shape of normalized fluorescence spectrum of (**A**) 4αZnPc^4+^ and (**B**) 4αβZnPc^4+^ in PBS with 10% *v*/*v* FBS.

A comparison of the spectra presented in Figure 2 indicated that an increase in the concentration of aryloxy-substituted Zn(II) phthalocyanines results in the broadening of their main fluorescence band and a shift in their emission maximum to longer wavelengths. These effects become particularly evident at a concentration of 100 μM for 4αβZnPc^4+^ with an asymmetric distribution of charged groups. In the spectrum of 4αβZnPc^4+^ at 100 μM, the intensity of the long-wave peak in the spectral range of 760–780 nm, corresponding to the aggregate forms of 4αZnPc^4+^, and, accordingly, its contribution to the integrated fluorescence intensity increases significantly [27]. Symmetric non-peripheral substitution reduces the effect of aggregation on the phthalocyanine spectra [23].

**Figure 3:**
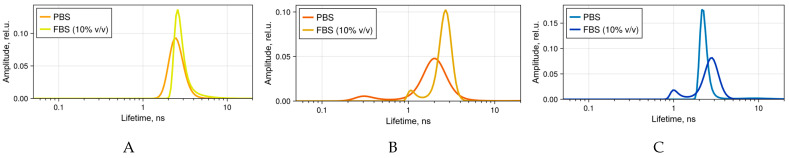
Fluorescence lifetimes distribution of (**A**) 4αZnPc^4+^, (**B**) 4αβZnPc^4+^, and (**C**) ZnPcChol^8+^ solutions in PBS or in PBS with FBS 10% (*v*/*v*) obtained using Maximum Entropy Method. Resolvable peaks were considered as individual components of the fluorescence decay. The concentration of phthalocyanines was 10 μM, except for 4αβZnPc^4+^ in PBS, where it was increased to 100 μM.

The results of these studies align with the observation of fractions with different fluorescence lifetimes of 4αZnPc^4+^, 4αβZnPc^4+^, and ZnPcChol^8+^ in PBS or PBS with FBS 10% (*v*/*v*) (Figure 3). In PBS, the main primary fluorescence fraction had the lifetime of 2.57 and 2.29 ns for 4αZnPc^4+^and 4αβZnPc^4+^, respectively. These values coincide with the fluorescence lifetime of water-soluble ZnPcChol^8+^ (2.29 ns), are typical for metal Pcs [28], and reflect the presence of non-aggregated components. In PBS with FBS 10% (*v*/*v*), this fluorescence lifetime slightly increased for 4αZnPc^4+^, 4αβZnPc^4+^, and ZnPcChol^8+^ (2.9, 2.7, 2.8, respectively). The second fraction with a shorter lifetime (Figure 3B,C) was found in 4αβZnPc^4+^ solutions in PBS (0.34 ns) and in 4αβZnPc^4+^ (1.15 ns) and ZnPcChol^8+^ (1.07 ns) solutions in PBS with FBS 10% (*v*/*v*). This fraction is presumably associated with the aggregated state of the dye molecules.

**Figure 4:**
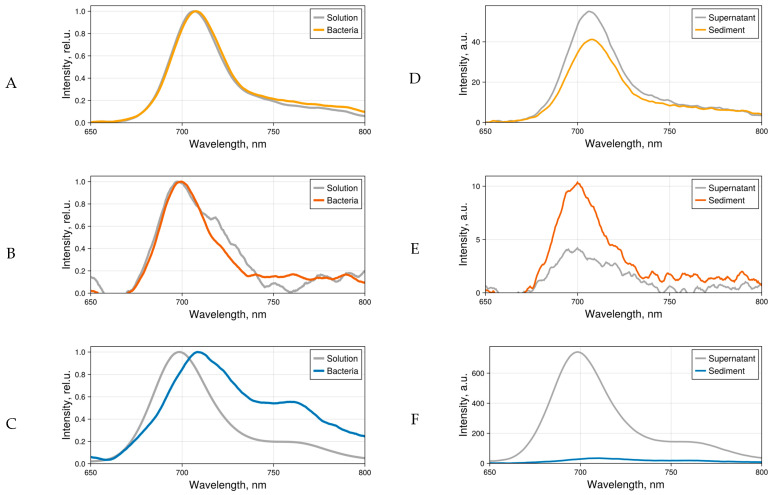
Normalized fluorescence spectra (**A**–**C**) and fluorescence spectra (**D**–**F**) of 4αZnPc^4+^ (**A**,**D**), 4αβZnPc^4+^ (**B**,**E**), and ZnPcChol^8+^ (**C**,**F**) in bacterial sediment and supernatant medium (PBS) after addition of phthalocyanine derivatives (5 µM).

In subsequent bacterial experiments, to avoid aggregation and at the same time obtain a sufficient fluorescence signal, the phthalocyanine concentration of 5 μM in PBS was used. When bacteria were added to 4αZnPc^4+^, 4αβZnPc^4+^, and ZnPcChol^8+^ solutions and subsequently precipitated, the sediment exhibited characteristic Pcs fluorescence (Figure 4A–C). In absolute terms, the fluorescence intensity of asymmetric 4αβZnPc^4+^ was extremely low, with an order of magnitude lower than that of symmetric 4αZnPc^4+^ (Figure 4D). In the bacterial sediment, fluorescence decreased slightly in the case of 4αZnPc^4+^ and increased twofold in the case of 4αβZnPc^4+^ (Figure 4E). Apparently, when 4αβZnPc^4+^ binds to bacterial membranes, its aggregates dissociate, which results in the increased fluorescence. Previously, dissociation of aggregates on the surface of bacteria was observed for cationic zinc phthalocyanines (ZnPc(Lys)_n_) [29]. In the presence of bacteria, the intensive fluorescence of ZnPcChol^8+^ was quenched (Figure 4F), and when measured in the sediment, it approximately matched that of 4αZnPc^4+^ and 4αβZnPc^4+^.

### 2.2. Fluorescence Microscopy Images

Fluorescence microscopy images of *E. coli* K12 TG1 cells incubated with or without polycationic Pcs were obtained using a scanning confocal microscope with laser excitation at 633 nm. In this work, the confocal microscope was used as a source for fluorescence excitation and as a tool for distinguishing fluorescence emitted by bacteria that were not fixed to glass from the fluorescence of Pcs in solution.

The bacterial cell emission in the Pcs-free control samples was only 1.2-fold higher than that from the surrounding medium. In the presence of symmetric 4αZnPc^4+^, the fluorescence contrast between bacteria and solution was 3.6 ± 1.2 (Table 1 shows data for five individual bacteria). In the presence of asymmetrically aryloxy-substituted 4αβZnPc^4+^, the contrast was even higher, with the fluorescent signal from the cells exceeding the signal from the surrounding medium by 7.3 ± 3.0. This indicates binding of aryloxy-substituted Pcs to the cell surface of Gram-negative bacteria *E. coli* K12 TG1. For a well-characterized antimicrobial photosensitizer, octacationic ZnPcChol^8+^ [30], which served as a comparison substance in our experiments, this parameter was 4.9 ± 2.4. ZnPcChol^8+^ readily binds to bacterial cells, and as we calculated for *E. coli*, when the binding sites on the cell surface are fully occupied, there are about a million molecules of ZnPcChol^8+^ present per cell [17]. Although the fluorescence of ZnPcChol^8+^ is quenched in the bound form, this quenching is compensated by the high local concentration of the bound fluorophore.

**Figure 5:**
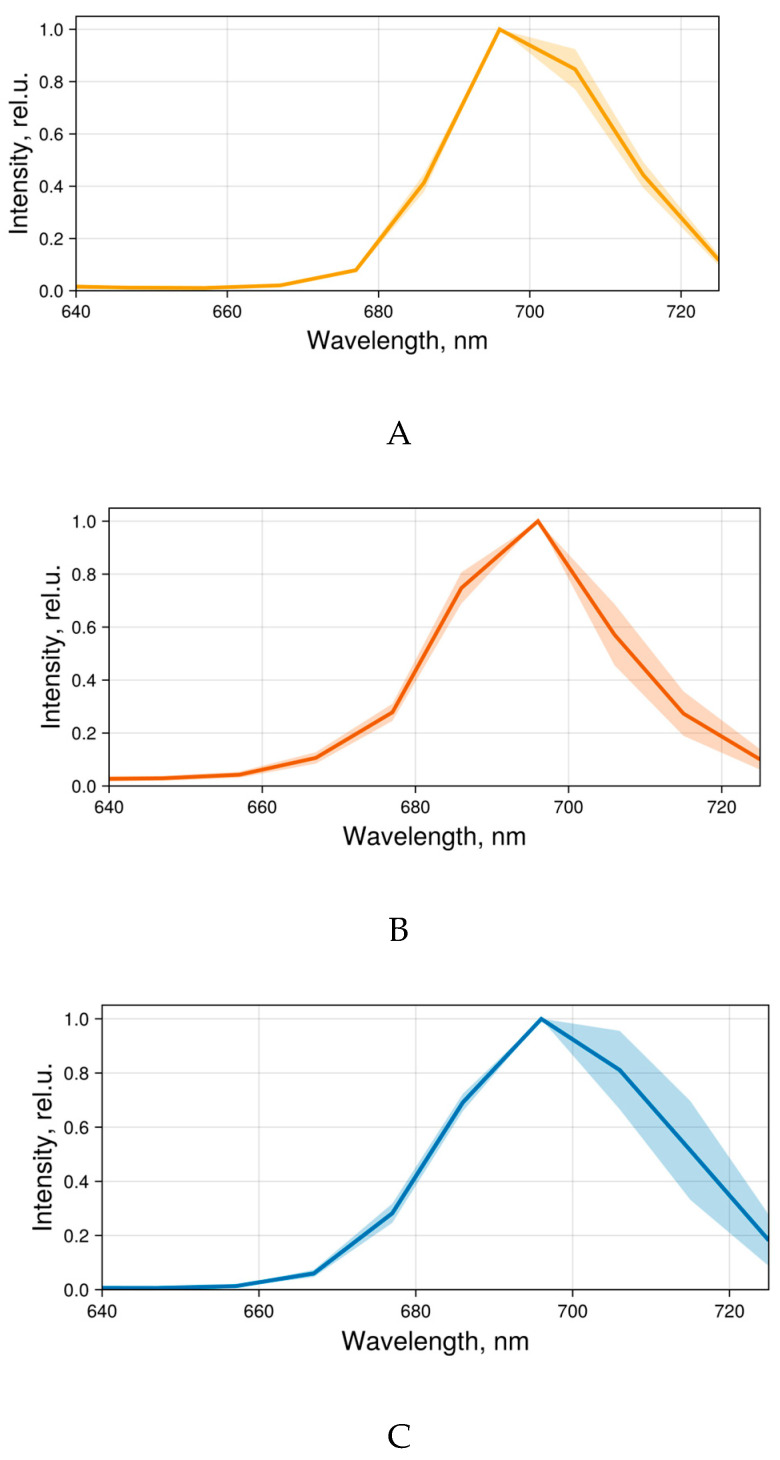
Averaged normalized fluorescence spectra of (**A**) 4αZnPc^4+^, (**B**) 4αβZnPc^4+^, and (**C**) ZnPcChol^8+^, bound to bacteria (N = 5), obtained using Zeiss laser scanning confocal microscope LSM-710 under laser excitation at 633 nm.

The fluorescence spectra of 4αZnPc^4+^, 4αβZnPc^4+^, and ZnPcChol^8+^ bound to bacteria were registered using the spectral resolution regime of the confocal microscope (Figure 5). The fluorescence spectra of symmetric tetra- and octacationic 4αZnPc^4+^ and ZnPcChol^8+^ typically exhibit a broad peak in the range of 696–706 nm. In the spectra of asymmetric 4αβZnPc^4+^, the fluorescence peak shifts to 686–696 nm. A shorter-wavelength maximum in the fluorescence spectrum of this compound was previously noted in bacterial sediment (Figure 4B).

### 2.3. Influence of Polycationic Phthalocyanines on Zeta Potential of Bacterial Cells

The binding of polycationic dyes to the negatively charged surface of bacterial cells can be detected by measuring changes in the electrophoretic mobility of the cells and, based on this, their zeta potential. In the buffer medium (10-fold diluted PBS with 0.01–0.02% DMSO) in control samples without the addition of phthalocyanine, the zeta potential of *E. coli* K12 TG1 was −37 mV. The addition of phthalocyanines at concentrations of 1 or 2 μM resulted in a concentration-dependent shift in the zeta potential towards more neutral values (Table 2). The magnitude of such shifts was significant, reaching 24.3 mV for 4αZnPc^4+^ and 21.3 for ZnPcChol^8+^ at 2 μM. The asymmetrically substituted 4αβZnPc^4+^ at 2 μM caused a shift of 18.2 mV. The obtained data clearly demonstrate the ability of all the studied polycationic Pcs to stain *E. coli* K12 TG1 cells. All three polycationic Pcs electrostatically bind to Gram-negative bacterial cells. The fact that 4αβZnPc^4+^, with a greater tendency to aggregate in water solutions, effectively neutralizes the surface of bacterial cells indicates the possibility of using this type of phthalocyanine derivative for the purpose of PDI.

### 2.4. Testing the Antibacterial Photodynamic Activity of Phthalocyanine Derivatives

Gram-negative pathogens possess a wider range of mechanisms of intrinsic and acquired resistance compared to Gram-positive pathogens, and are capable of acquiring and developing resistance to many classes of antibiotics. This renders them a priority for the development of novel methods of antimicrobial therapy [31]. The photodynamic potential of phthalocyanine derivatives against Gram-negative bacteria was studied using *E. coli* K12 TG1. This genetically modified strain, with a cloned complete lux operon from the luminescent soil entomopathogenic bacterium Photorhabdus luminescens ZMI, is a useful tool for screening photosensitizers for PDI/APDT [32]. The method is based on the correlation between photosensitized quenching of bioluminescence and the rate of inactivation of colony-forming units (CFU) in the test culture [33].

**Figure 6:**
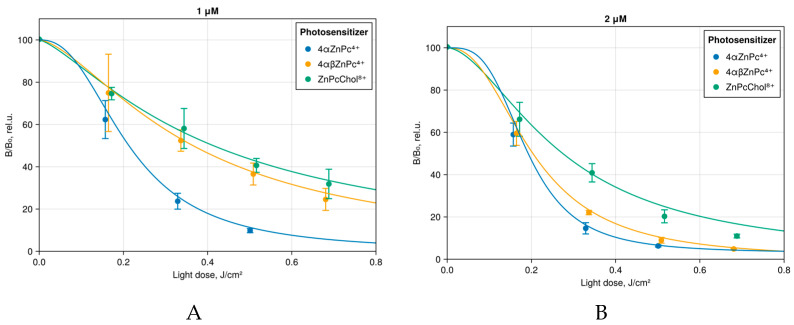
Bioluminescence of *E. coli* K12 TG1 (luxABCDE) after 10 min incubation with the studied photosensitizers at (**A**) 1 μM and (**B**) 2 μM concentration, and further irradiation with red light-emitting diode (LED) 686 nm.

Aryloxy-substituted Zn(II) Pcs 4αZnPc^4+^ and 4αβZnPc^4+^ have been shown to be effective generators of ROS with quantum yields for singlet oxygen as high as 0.65–0.69 [23,24]. The destructive effect of ROS on plasma membranes, which are the primary targets of bacterial PDI, is based on the oxidation of unsaturated lipid acids, leading to a disruption of the barrier properties of the membrane with the formation of defects through which the loss of intracellular components occurs, which ultimately leads to cell death [18]. The plasma membrane is separated from the environment by a cell wall, the structure and permeability of which to photosensitizers differ between Gram-positive and Gram-negative bacteria. This determines the difference in the sensitivity of these two groups of bacteria to PDI [34]. Anionic photosensitizers demonstrate adequate efficacy in photodynamic inactivation of Gram-positive bacteria, but their efficiency is suboptimal for Gram-negative and mixed microbiota. Polycationic photosensitizers are quite effective against both types of bacteria and their biofilms. Indeed, both 4αZnPc^4+^ and 4αβZnPc^4+^ induced inhibition of *E. coli* K12 TG1 (luxABCDE) bioluminescence (Figure 6). Notably, 4αZnPc^4+^ and 4αβZnPc^4+^ exhibited comparable efficacy in PDI to the water-soluble monomeric ZnPcChol^8+^, a photosensitizer with proven antibacterial activity.

## 3. Materials and Methods

### 3.1. Phthalocyanine Derivatives

Long-wavelength aryloxy-substituted tetracationic phthalocyanines (Figure 1) with excitation in the spectral range of 680–690 nm were used in the study: a symmetric non-peripheral substituted 4αZnPc^4+^—1,8(11),15(18),22(25)-tetrakis(4-((diethylmethyl-ammonium)methyl)phenoxy) zinc phthalocyaninate tetraiodide and an asymmetric 4αβZnPc^4+^ with all four substituents attached to one benzene ring—1,2,3,4-tetrakis(4-((diethylmethyl-ammonium)methyl)phenoxy) zinc phthalocyaninate tetraiodide, synthesized at Frumkin Institute of Physical Chemistry and Electrochemistry [23,33]. Octacationic ZnPcChol^8+^ zinc octakis(cholinyl)phthalocyanine, developed at the Institute of Organic Intermediates and Dyes (Dolgoprudny, Russia) [22], was used in this study as a comparison compound.

4αZnPc^4+^ was prepared according to standard procedure by template cyclotetramerization of 3-(4-(diethylaminomethyl)phenoxy)phthalonitrile with zinc acetate in boiling 1-pentanol in the presence of 1,8-Diazabicyclo[5.4.0]undec-7-ene (DBU), followed by quaternization in chloroform [24].

4αβZnPc^4+^ was synthesized by template cyclotetramerization of 1,2,3,4-tetrakis(4-(diethylaminomethyl)phenoxy)phthalonitrile and unsubstituted phthalonitrile with magnesium acetate in boiling 1-pentanol in the presence of DBU. This mixture was then treated with CF_3_CO_2_H/H_2_O in chloroform. This resulted in a mixture of free phthalocyanine bases, which were then converted into a mixture of zinc complexes (zinc acetate, N,N-dimethylformamide, 110 °C). 1,2,3,4-tetrakis(4-(diethylaminomethyl)phenoxy)zinc phthalocyaninate was isolated by size-exclusion chromatography on Bio-Beads SX-1 and quaternized in chloroform, yielding the target 1,2,3,4-tetrakis(4-(diethylammoniumomethyl)phenoxy)zinc phthalocyaninate tetraiodide [23].

Spectral data for these complexes (1H-NMR and UV-Vis) are given in Supporting Information (Table S1).

Stock solutions (10 mM) of 4αZnPc^4+^ and 4αβZnPc^4+^ were prepared in dimethyl sulfoxide (DMSO) and subsequently transferred to phosphate-buffered saline (PBS), which was diluted tenfold with distilled water (pH 7.4) to obtain a phthalocyanine concentration of 100 μM in the presence of 1% DMSO. These solutions were used for further experiments. Water-soluble ZnPcChol^8+^ was prepared directly in PBS, diluted tenfold with distilled water (pH 7.4).

### 3.2. Fluorescence

The shape of the fluorescence spectra and the fluorescence intensity of polycationic phthalocyanines were studied using the LESA-01-BIOSPEC spectrometer (Biospec, Moscow, Russia) under excitation by a 633 nm laser. The data were analyzed with UnoMomento software v.2.1.3 (Biospec, Moscow, Russia). In order to enhance the luminescence of aryloxy-substituted phthalocyanines in solutions in these experiments, 10% (*v*/*v*) FBS was added.

In order to estimate the fluorescence lifetime, phthalocyanines were excited by picosecond laser pulses with a wavelength of 637 nm, and the fluorescence was recorded using a spectroscopic complex with a streak camera (Hamamatsu Photonics, Hamamatsu, Japan).

### 3.3. Biosensor Escherichia coli

A genetically engineered strain of Gram-negative bacteria *E. coli* K12 TG1 (luxABCDE) was used as a biosensor [32,33]. Prior to the experiment, the bacterial culture (about 3 × 10^7^ CFU/mL) was suspended in PBS buffer (pH 7.4), diluted tenfold with distilled water PBS/10 (pH 7.4), with the addition of 10 μM CaCl_2_, and maintained for 1 h at 20–22 °C to achieve stable bioluminescence. Photodynamic inactivation (PDI) was assessed by the decrease in the bioluminescence intensity of a bacterial cell suspension (2 mL) irradiated after 10 min of preincubation at 20–22 °C in the absence of illumination with one of the phthalocyanines, compared with the bioluminescence intensity of cells irradiated with the same dose of light but without adding a photosensitizer. Bioluminescence quenching was expressed via ((B_0_ − B)/B_0_), where B_0_ is the bioluminescence intensity of cells irradiated with red light in the absence of dye, and B is the bioluminescence intensity of cells irradiated in the presence of dyes. The intensity of bioluminescence was measured using a Sirius Smart Line TL luminometer (Berthold GmbH & Co., Bad Wildbad, Germany). The inaccuracy of the method does not exceed 10%.

### 3.4. Fluorescence Microscopy of Biosensor E. coli with the Studied Photosensitizers

Fluorescence microscopy images were obtained using a Zeiss laser scanning confocal microscope LSM-710 (Carl Zeiss AG, Oberkochen, Germany) under laser excitation at 633 nm, both in a single-channel mode and with spectral resolution. Bacterial suspensions (about 10^8^ CFU/mL) were prepared similarly to those used for photosensitization. Following the addition of one of the phthalocyanines at a final concentration of 5 μM, the bacterial suspension was incubated in the dark at room temperature for 10 min.

### 3.5. Zeta Potential

The surface charge of *E. coli* K12 TG1 (luxABCDE) cells was assessed based on the zeta potential values measured using a Zetasizer Nano ZS analyzer (Malvern Instruments, Worcestershire, UK). The zeta potential of bacterial suspensions was measured using methods analogous to those described previously for bioluminescent measurements. The electrostatic binding of phthalocyanines to bacterial cells was determined by neutralizing the negative zeta potential, that is, a shift in the zeta potential values towards more electroneutral values.

All experiments on bioluminescence and zeta potential were performed in triplicate. The measurement of fluorescence intensity was conducted for a minimum of five bacterial samples. The figures show individual fluorescence spectra and, in the case of bioluminescence and zeta potential parameters, averaged data with standard deviations.

## 4. Conclusions

The obtained results allow us to conclude that the studied tetracationic aryloxy-substituted Zn(II) phthalocyanines effectively bind to the oppositely charged cell wall of Gram-negative bacteria *E. coli*. It is confirmed by both the results of the cell’s zeta potential neutralization in the presence of phthalocyanine derivatives and fluorescence microscopy images. It has been demonstrated that the binding of phthalocyanine results in a contrast of the emission of the stained bacterial cells compared to that of the solution. The aggregation of phthalocyanine molecules in a water medium, which is more pronounced in the case of an asymmetrically substituted derivative, has little effect on the photodynamic properties of the studied tetracationic phthalocyanines. Both symmetric and asymmetric aryloxy-substituted phthalocyanines demonstrated high activity in the bacterial bioluminescence assay and have significant potential for further studies as antibacterial photosensitizers.

## Figures and Tables

**Table 1:** Ratio between the mean fluorescence of bacteria (N = 5) and the mean fluorescence of the surrounding solution after 10 min incubation with 5 µM of different phthalocyanine derivatives obtained using a scanning confocal microscope.

N	4αZnPc^4+^	4αβZnPc^4+^	ZnPcChol^8+^
1	4.38	7.23	5.08
2	3.12	6.01	1.63
3	5.05	6.42	2.51
4	3.57	13.06	5.20
5	1.90	7.25	8.14

**Table 2:** The zeta potential of *E. coli* K12 TG1 biosensor cells. Initial value without incubation with 4αZnPc^4+^, 4αβZnPc^4+^, and ZnPcChol^8+^ was −37.0 (±3.8) mV. The deviation of the zeta potential is indicated in brackets.

Phthalocyanines (Pcs)	Pcs Concentration, 1 µM	Pcs Concentration, 2 µM
4αZnPc^4+^	−28.6 (±6.8)	−12.7 (±5.4)
4αβZnPc^4+^	−25.7 (±4.9)	−19.8 (±7.8)
ZnPcChol^8+^	−24.8 (±5.7)	−15.7 (±6.1)

## Data Availability

The raw data supporting the conclusions of this article will be made available by the authors on request.

## Supplementary Materials

The following supporting information can be downloaded at: https://www.mdpi.com/article/10.3390/ijms26199414/s1.
