# Supplementary material for: The Photodynamic Antibacterial Potential of New Tetracationic Zinc(II) Phthalocyanines Bearing 4-((Diethylmethylammonium)methyl)phenoxy Substituents
Source: Int J Mol Sci. 2025 Sep 26;26(19):9414. doi: 10.3390/ijms26199414 (PMC12524610; doi:10.3390/ijms26199414)
Supplement: Supplementary file 1 [file ijms-26-09414-s001.zip › ijms-3819938-supplementary.pdf]

International Journal of Molecular Sciences

Table S1. Spectral data for 4αZnPc^{4+} and 4$\alpha\beta$ZnPc^{4+}	
**4αZnPc^{4+}**	
**1H NMR** (600 MHz, DMSO-*d6*)	δ 9.46 (dd, *J* = 12.2, 7.4 Hz, H_{Pc}), 9.29 (dd, *J* = 7.4, 3.6 Hz, H_{Pc}), 8.80 (dd, *J* = 23.1, 7.5 Hz, H_{Pc}), 8.64 (dd, *J* = 27.0, 7.3 Hz, H_{Pc}), 8.40 (t, *J* = 7.5 Hz, H_{Pc}), 8.23 (dt, *J* = 19.4, 7.5 Hz, H_{Pc}), 8.09–7.99 (m), 7.96 (t, *J* = 8.0 Hz, H_{Pc}) (10H), 7.66–7.49 (m, 16H, H_{OAr}), 7.37 (dd, *J* = 8.4, 4.9 Hz), 7.30 (dd, *J* = 8.5, 3.8 Hz) (2H, H_{Pc}), 4.55–4.52 (m), 4.45 (dd, *J* = 14.1, 8.1 Hz) (8H, H_{Bn}), 3.21 (ddt, *J* = 25.4, 18.5, 5.9 Hz), 3.10 (ddt, *J* = 20.9, 14.7, 7.3 Hz) (16H, $H_{CH2(Et)}$), 2.90–2.84 (m), 2.74–2.70 (m) (12H, $H_{Me(N)}$), 1.32 (ddd, *J* = 13.9, 8.3, 5.3 Hz), 1.22–1.16 (m, 24H, $H_{Me(Et)}$).
**UV–Vis** (DMSO, 5 μM)	λ_{max}/nm (log ε): 334 (4.55), 373 (4.53), 624 (4.50), 693 (5.26)
**4$\alpha\beta$ZnPc^{4+}**	
**1H NMR** (600 MHz, DMSO-*d6*)	δ 9.35 (s, 4H, H_{Pc}), 8.72 (d, *J* = 7.4 Hz, 2H, H_{Pc}), 8.27–8.19 (m, 4H, H_{Pc}), 8.15 (t, *J* = 7.2 Hz, 2H, H_{Pc}), 7.58 (d, *J* = 8.4 Hz, 4H, H_{OAr}), 7.50 (t, *J* = 9.3 Hz, 8H, H_{OAr}), 7.39–7.32 (m, 4H, H_{OAr}), 4.48 (s, 4H, H_{Bn}), 4.39 (s, 4H, H_{Bn}), 3.29–3.22 (m, 4H, $H_{CH2(Et)}$), 3.18 (dd, *J* = 8.6, 5.9 Hz, 4H, $H_{CH2(Et)}$), 3.04 (dd, *J* = 13.6, 7.0 Hz, 4H, $H_{CH2(Et)}$), 2.90 (dd, *J* = 13.5, 7.0 Hz, 4H, $H_{CH2(Et)}$), 2.80 (s, 6H, $H_{Me(N)}$), 2.52 (s, 6H, $H_{Me(N)}$), 1.33 (t, *J* = 7.3 Hz, 12H, H_{Me}), 1.05 (t, *J* = 7.2 Hz, 12H, H_{Me}).
**UV–Vis** (DMSO, 1 μM)	λ_{max}/nm (log ε): 350 (4.80), 613 (4.47), 634 (4.48), 674 (5.09), 690 (5.21)
